# Development of a Transdermal Delivery System for Tenofovir Alafenamide, a Prodrug of Tenofovir with Potent Antiviral Activity Against HIV and HBV

**DOI:** 10.3390/pharmaceutics11040173

**Published:** 2019-04-09

**Authors:** Ashana Puri, Sonalika A. Bhattaccharjee, Wei Zhang, Meredith Clark, Onkar N. Singh, Gustavo F. Doncel, Ajay K. Banga

**Affiliations:** 1 Center for Drug Delivery Research, Department of Pharmaceutical Sciences, College of Pharmacy, Mercer University, Atlanta, GA 30341, USA; ashana.puri@live.mercer.edu (A.P.); sonalika.arup.bhattaccharjee@live.mercer.edu (S.A.B.); 2 CONRAD, Department of Obstetrics and Gynecology, Eastern Virginia Medical School, Arlington, VA 22209, USA; wzhang@conrad.org (W.Z.); mclark@conrad.org (M.C.); osingh@conrad.org (O.S.); gfdoncel@conrad.org (G.F.D.)

**Keywords:** transdermal patch, tenofovir alafenamide, acrylate adhesive, in vitro permeation, silicone adhesive, suspension patch

## Abstract

Tenofovir alafenamide (TAF) is an effective nucleotide reverse transcriptase inhibitor that is used in the treatment of HIV-1 and HBV. Currently, it is being investigated for HIV prophylaxis. Oral TAF regimens require daily intake, which hampers adherence and increases the possibility of viral resistance. Long-acting formulations would significantly reduce this problem. Therefore, the aim of this study was to develop a transdermal patch containing TAF and investigate its performance in vitro through human epidermis. Two types of TAF patches were manufactured. Transparent patches were prepared using acrylate adhesive (DURO-TAK 87-2516), and suspension patches were prepared using silicone (BIO-PSA 7-4301) and polyisobutylene (DURO-TAK 87-6908) adhesives. In vitro permeation studies were performed while using vertical Franz diffusion cells for seven days. An optimized silicone-based patch was characterized for its adhesive properties and tested for skin irritation. The acrylate-based patches, comprising 2% w/w TAF and a combination of chemical enhancers, showed a maximum flux of 0.60 ± 0.09 µg/cm^2^/h. However, the silicone-based patch comprising of 15% w/w TAF showed the highest permeation (7.24 ± 0.47 μg/cm^2^/h). This study demonstrates the feasibility of developing silicone-based transdermal patches that can deliver a therapeutically relevant dose of TAF for the control of HIV and HBV infections.

## 1. Introduction

Tenofovir (TFV) represents the cornerstone of prophylactic and therapeutic approaches to control HIV infection [1]. Tenofovir alafenamide fumarate (TAF), a prodrug, is currently replacing TFV disoproxil fumarate, which shows better affinity for lymphoid tissue, yielding higher levels of TFV-diphosphate (active metabolite) and lower levels of plasma TFV, and displaying a better renal and bone safety profile [2]. Due to these advantages, TAF is also being considered in the treatment of chronic HBV infection [1]. For HIV, TAF is typically administered, in combination with other antiretrovirals (ARVs), in daily single tablet regimens (STRs). Although STRs represent a significant advantage over the need to take multiple tablets three times a day, which characterized the beginnings of highly active antiretroviral therapy, it still remains a high burden to the patient, leading to poor adherence, lower efficacy, and increased risk of viral resistance. Therefore, sustained release of ARVs, which can reduce these problems, is needed. The development of sustained release delivery technologies in this space would be of high clinical relevance.

A variety of delivery systems, such as tablets (oral), tablet-like inserts (vaginal/rectal), gels (rectal/vaginal), vaginal films, ointments, rings, diaphragm based devices and electrospun fibers, oral/parenteral liposomes and nanoparticles, injectables, and subdermal implants for prophylactic or therapeutic anti-HIV agents have been explored [3,4,5,6,7,8,9]. Drug delivery systems, such as daily oral tablets and coitally-dependent vaginal gels, are typically short-acting and they have been associated with poorer user adherence. Long-acting coitally-independent systems, such as implants and injectables, do not present these problems, but are riskier if safety or idiosyncratic issues arise [7,10,11,12,13]. An intermediate duration drug delivery system, such as a transdermal patch, exerting its pharmacological effect for a few days to a week, would thus be beneficial to the users, providing a more sophisticated and convenient therapy option.

Transdermal drug delivery offers an attractive alternative to oral and other systemic routes of delivery, allowing for the drug substances to reach the systemic circulation across the skin barrier. Transdermal drug delivery systems can be beneficial in preventing the hepatic first pass effect of drugs, eliminating peak and valley plasma drug concentrations, which are usually associated with the oral and injectable drug delivery, avoiding the degradation of drugs in GI tract, and, in general, being non-invasive and convenient to administer [14]. ARV drug substances may potentially be delivered through the transdermal route in a consistent and sustained manner (e.g., zero order release kinetics), which would provide several advantages over the other delivery systems that have been explored so far [15]. Being non-invasive and much easier to apply, without the intervention of a health care provider, transdermal patches may provide more user adherence than the injectable or implantable delivery systems that are currently under investigation. Additionally, transdermal patches can be easily discontinued if the need arises, making them safer to use. The aim of this study was to develop and characterize transdermal patches for the delivery of ARV agents, in particular TAF, and investigate its permeation through human epidermis.

There are generally two types of passive transdermal patches, namely, matrix (drug-in-adhesive) and reservoir. The matrix-based transdermal patch, which was the system of choice in this study, consists of a release liner, an adhesive matrix, and an impermeable backing membrane. In addition to the ease of use and manufacturability, as well as the acceptable cost of goods, one of the significant advantages of matrix type transdermal patches when compared to reservoir patches is the absence of dose dumping [16]. The matrix transdermal patches are usually prepared using organic solvent based pressure sensitive adhesives (PSAs), such as acrylate copolymer, silicone, polyisobutylene (PIB), either alone or in combination with each other [17]. Drug substance can be either dissolved or dispersed in the adhesive matrix, resulting in clear/transparent/translucent or suspension patches, respectively.

From a transdermal patch development perspective, drugs with low molecular weight (<500 Da), suitable melting point (<250 °C), and moderate log P (1–3) are ideal for passive permeation through skin. With a molecular weight of 476.47 g/mol, melting point of 279 °C, and having a logP =1.8, as calculated using chemicalize software (MarvinSketch: version 6.2.2, ChemAxon, Hungary, Europe), TAF, the newest prodrug of TFV in the market, is a promising ARV candidate for the development of transdermal patch formulation. TAF is commercially available with oral daily doses of 10 mg and 25 mg [18]. When considering the higher dose of 25 mg and low bioavailability of orally administered TAF (25%) vs transdermal, 6 mg of it would be needed in the systemic circulation [19,20]. However, 8 mg/day was established as the target dose to be on the safer side and keeping a window of deviations.

The aim of this study was to develop a transdermal patch for sustained release of TAF for approximately one-week duration. To our knowledge, transdermal patches comprising of ARV agents for HIV therapy or prophylaxis have not yet been developed. Thus, this is the first study to report such a system. In the present study, transparent acrylate based patches, as well as silicone and PIB based suspension patches of various compositions, were prepared and evaluated for in vitro permeation across human epidermis for seven days. In order to release a TAF dose of 8 mg/day from a 50 cm^2^ transdermal patch, the targeted permeation flux was about 7 µg/cm^2^/h. Different chemical enhancers, plasticizers, as well as crystallization inhibiting agents, were included in the patch formulations. Patches were evaluated for stability, skin irritation, and physical characteristics, such as tack, peel, and shear adhesion.

## 2. Materials and Methods

### 2.1. Materials

TAF (free base, CAS number: 379270-37-8) was purchased from Pharmacodia Co., LTD (Beijing, China). Backing membranes (Scotchpak^TM^ 9733-2.05 mil polyester film; CoTran™ 9702, 9706, 9728- ethylene vinyl acetate copolymer (EVAC); CoTran™ 9718, 9720 – polyethylene film); and, release liner (Scotchpak^TM^ 1022- 3 mil fluoropolymer coated polyester film) were gifted by 3M (St. Paul, MN, USA). Silicone coated poly-ethylene terephthalate (PET) films (1 mil): 48101-4400B/000, 44916-7300AM/000, and 40987-U4162/000 were procured from Loparex (Cary, NC, USA). Acrylate PSA (DURO-TAK 87-2516) as well as PIB adhesive (DURO-TAK 87-6908) were obtained as gift samples from Henkel Corporation (Dusseldorf, Germany). The Dow Corning Corporation provided Silicone adhesive (BIO-PSA 7-4301) provided as a gift sample (Washington, DC, USA). Mineral oil, oleyl alcohol, polyethylene glycol (PEG), and polyvinyl pyrrolidone (PVP 360) were obtained from Sigma–Aldrich (St. Louis, MO, USA). Propylene glycol (PG) and oleic acid (OA) were purchased from Ekichem (Joliet, IL, USA) and Croda Inc. (Edison, NJ, USA), respectively. Triacetin, octisalate, gentamycin sulfate, tetra hydrogen furan (THF), sodium dihydrogen phosphate, sodium hydroxide, and phosphate buffered saline, pH 7.4 (PBS) were obtained from Fisher Scientific (Pittsburgh, PA, USA). Kollidon^®^ VA64, Kollidon^®^ 90F, and Kollidon^®^ 30LP were purchased from BASF (Florham Park, NJ, USA). Methanol, ethanol, acetonitrile, and trifluoroacetic acid were purchased from Pharmco-aaper (Brookfield, CT, USA). Dermatomed human cadaver skin was obtained from New York Fire Fighters (New York, NY, USA).

### 2.2. Methods

#### 2.2.1. Slide Crystallization Studies

TAF was dissolved in methanol (10 mg/mL) and a drop of this solution was placed on a glass slide. Methanol was allowed to evaporate at room temperature (RT) under a fume hood. The drug crystals that were obtained were then observed under Leica DM 750 optical microscope (Leica Microsystems Inc., Buffalo Grove, IL, USA). The DFC-295 camera, which was attached to the microscope, was used to capture images at magnifications of 10× or 20× (as specified). Similar procedure was used to determine the saturation solubility of TAF in different adhesives (DURO-TAK 87-2516, DURO-TAK 87-6908, and BIO-PSA 7-4301), solution of 5% (*w*/*w*) OA in DURO-TAK 87-2516, as well as in the additives: PVP 360, Kollidon^®^ VA64, Kollidon^®^ 90F, and Kollidon^®^ 30LP. For solubility studies in adhesives, blends of different concentrations of TAF in adhesives, ranging from 1–12% *w*/*w* (dry weight) were prepared and a drop of the transparent blends (with completely dissolved drug) was individually placed on glass slides and kept in a flameproof oven at 100 °C for 30 min. for the evaporation of the solvents in the adhesives. Similarly, different blends with TAF concentration, ranging from 3–5% (*w*/*w*) in a mixture of 5% (*w*/*w*) OA in DURO-TAK 87-2516 (dry weight basis), were prepared and a drop of each of the solutions was placed on the glass slides and then exposed to the same drying conditions, as mentioned above. In order to determine the solubility of TAF in different additives, the two components were weighed in the following ratios (% *w*/*w*): 1:9, 2:8, 3:7, 4:6, 5:5, 6:4, 7:3, 8:2, 9:1, and then dissolved in methanol. A drop of each of these solutions was individually mounted on glass slides and observed for crystals after allowing for the evaporation of methanol at RT. The appearance of crystals in all of the test samples was observed for a week before concluding the saturation solubility of TAF in different components. The latter was indicated by the highest concentration at which no crystals were observed [21,22]. The percentages of solid content that were used to calculate the wet weight of adhesives were 41.5% for DURO-TAK 87-2516, 38% for DURO-TAK 87-6908, and 60% for BIO-PSA 7-4301.

#### 2.2.2. Preparation of Drug in Adhesive Patches of TAF

##### Formulation of Acrylate-Based Patches

Pre-determined amounts of TAF, adhesives, and additives (OA, oleyl alcohol, PG, octisalate, triacetin, as specified in Table 1) were weighed into glass jars with airtight lids to minimize the loss of organic solvents. For the patch formulations AP4 to AP11, excipients, such as PVP 360, Kollidon^®^ VA64, Kollidon^®^ 90F, and Kollidon^®^ 30LP were separately dissolved in methanol and the solutions were then added to the mixture of adhesive and other excipients. The blends were kept overnight on the rotary mixer (Preiser Scientific Inc., St. Albans, WV, USA). Table 1 shows the compositions of the different acrylate based TAF patches. Solid content % of 41.5 was used for the calculation of the wet weight of DURO-TAK 87-2516 for the formulations. The homogenous mixtures were then casted on the release liner (fluoropolymer coated side of Scotchpak^TM^ 1022) using a Gardner film casting knife (BYK-AG-4300 series, Columbia, MD, USA). The target coat weight of different patches varied from 100–400 gsm (grams per square meter). The casted sheets were dried in a flameproof oven at 95 °C for 40 min. After drying, they were laminated using Scotchpak^TM^ 9733 (backing membrane) with the help of a roller, ensuring that no air pockets were formed. The 200 gsm and 400 gsm patches were made by preparing one 100 gsm patch and then stacking 1 and 3, 100 gsm cast films (coated on release liner), respectively, on that one by one, after removing the liner of the previous laminate.

##### Microscopy of Acrylate-Based Patches

The prepared acrylate patches were punched and stored at 40 °C, RT, and −20 °C, and then observed for the occurrence of crystallization under an optical microscope (Leica DM 750) at predetermined time points for up to six months [23]. In case of patches that showed the appearance of crystals in the first week of storage at RT or 40 °C, microscopic observations after storage at −20 °C were not performed.

##### Formulation of TAF Suspension Patches

The suspension patches of TAF were prepared using silicone (BIO-PSA 7-4301) and PIB (DURO-TAK 87-6908) adhesive. TAF was first levigated with mineral oil and other additives (OA and oleyl alcohol) were then added and mixed using Benchmixer^TM^ (Benchmark Scientific Inc., Edison, NJ, USA). Heptane was then added to the blend and the drug suspension was homogenized using a high-speed homogenizer (OmniTHQ, Omni International, NW, GA, USA). Heptane was then allowed to evaporate at 90 °C for 5 min. and the adhesive was added to the remaining mixture. The final blend was allowed to mix overnight, then homogenized for 1 min, and then casted on the release liner. It was dried in a flameproof oven and the casted film was then laminated with the backing membrane. Table 2 elaborates on the compositions, homogenization conditions, release liners, backing membranes, and drying conditions employed for the formulation of different TAF suspension patches.

###### Visual Observations of the TAF Suspension Patches

The suspension patches with varying composition and material components were prepared and observed for the following visual changes for about two weeks: phase separation, contraction/shrinkage of the film, residue on release liner after peeling, ease of peeling off the patches applied on human skin, as well as any residue on skin after removal of the patches.

###### Effect of Homogenization on Particle Size of TAF

The particle size of TAF, before and after homogenization, was determined using optical microscopy. For the former, pure TAF was dispersed in heptane and a drop of the same was placed on a glass slide and observed under optical microscope. For the size of TAF particles after homogenization, a drop of the suspension, before the addition of the adhesive, was mounted on glass slide and observed under the optical microscope. Size of particles (*n* = 50) was measured using ImageJ 1.41o software (National Institutes of Health, USA) and it has been reported as average ± SD.

#### 2.2.3. Coat Weight and TAF Content of the Patches

Punching and weighing 4.91 cm^2^ or 1 cm^2^ laminates (from different areas of the patch, *n* = 3) using analytical balance (Mettler Toledo, Columbus, OH, USA) and subtracting the weight of equal sized respective release liner and backing membrane from the same determined the coat weight of the prepared patches. For the determination of drug content in the patches, the punched patches were placed in 10 mL of THF after removal of the release liner and allowed to shake overnight. The solutions were then diluted ten times with methanol and centrifuged at 13,400 rpm for 10 min. The supernatants were then analyzed while using high performance liquid chromatography (HPLC) to quantitate the amount of TAF in the same. Comparing the experimental and theoretical drug content values used for processing indicated the stability of TAF after exposure to high temperature conditions. The latter were calculated based on the targeted coat weight for each laminate and the percentage of drug loaded (dry weight% when considering the solvent loss upon drying) in the formulation blend. Results have been reported as average ± SD.

#### 2.2.4. In Vitro Skin Permeation Studies

In vitro permeation studies of TAF from transdermal patches through human epidermis were performed for seven days using in vitro vertical Franz diffusion cells (PermeGear, Inc., Hellertown, PA, USA), providing an effective diffusion area of 0.64 cm^2^ (*n* = 6).

##### Separation of Epidermis

For each permeation study, human epidermis was freshly isolated from dermatomed human skin by the heat-separation method. Skin was immersed in 10 mM PBS, pH 7.4 for 2 min. at 60 °C. Epidermis was then carefully manually peeled off, with the help of forceps and spatula, and thereafter cut into pieces of suitable size for mounting on the Franz cells [24].

##### Epidermal Integrity and Thickness Assessment

Resistance of human epidermis was measured in order to select the pieces with acceptable initial barrier integrity for the in vitro permeation study [25]. This was carried out with the use of silver-silver chloride electrodes that were attached to a digital multimeter: 34410A 6 ½ (Agilent Technologies, CA, USA) as well as an arbitrary waveform generator (Agilent 33220A, 20 MHz Function). Epidermis was mounted on the Franz diffusion cells and left for equilibration for 15 min. Phosphate buffer, pH 6.0, was then added in the donor (300 µL) and receptor (5 mL), respectively. Following equilibration, silver and silver chloride electrodes were placed in the receptor and donor compartment, respectively. Load resistor (*R*_L_) attachment was in series with skin, and the voltage drop across the entire circuit and skin (*V*_S_) was displayed on the multimeter (*V*_O_). The following formula was used to measure the skin resistance (*R*_S_):

$$Rs=Vs\;R_{L}/\;(Vo\;-\;Vs)$$

where, *V*_O_ and *R*_L_ were 100 mV and 100 kΩ, respectively. Epidermis pieces with electrical resistance of more than 10 kΩ were selected for the permeation study. A thickness gauge measured the thickness of the selected epidermis pieces (Cedarhurst, NY, USA).

##### Selection of the Receptor Solution

The stability of TAF in different solvent systems (10 mM PBS, pH 7.4; phosphate buffer, pH 6.0; 10 mM PBS, pH 7.4: PEG 400 - 1:1; PEG 400) at 37 °C was assessed in order to select a suitable receptor solution for the seven day permeation studies. For this study, different concentrations of TAF were prepared (0.5, 5, 50 µg/mL) in the above specified solvents and kept at 37 °C. The solutions were analyzed for drug content at 0, and after 24 h using HPLC. The solubility of TAF in the selected receptor media (phosphate buffer, pH 6.0) was determined to ensure the maintenance of sink conditions. For this purpose, excess drug was added to 1.0 mL of the buffer and it was allowed to shake for 24 h at RT. Thereafter, the solution was filtered using 0.22 µm syringe filters (Cell treat Scientific Products, Shirley, MA, USA) and analyzed using HPLC after suitable dilution.

##### In Vitro Skin Permeation Set Up

The permeation of TAF from different transdermal patch formulations (donor), through human epidermis was investigated using the Franz diffusion cell set up as mentioned in Table 3. While taking the stability of TAF, as well as the maintenance of sink conditions, into consideration, phosphate buffer, pH 6.0 having gentamycin sulfate (80 mg/L) as an anti-microbial agent for the seven day permeation study [22], was selected as the receptor compartment. The temperature of the receptor phase was maintained at 37 °C and it was constantly stirred at 600 rpm. The freshly isolated epidermis pieces were clamped between the donor and receptor compartments. After measuring the skin resistance through the procedure described above, phosphate buffer from the donor was pipetted out, epidermis was removed, placed flat on a glass plate, and then dried with the help of Kimwipes. The release liners of the patches (of size enough to cover the diffusion area) were removed and the latter were applied carefully on the dried epidermis, such that the adhesive layer of the patch was adhered to the stratum corneum side of the skin. The glass rod was rolled on the patches to ensure their adherence to the skin. The epidermis pieces with the applied patches were then mounted between the donor and receptor compartment and the entire set up was secured in place using a clamp. Receptor (0.3 mL) was sampled at 0, 2, 4, 6, and 8 h with replacement with fresh receptor solution and entire receptor (5 mL) was removed and replaced with fresh buffer at 24, 48, 72, 96, 120, 144, and 168 h. All of the samples were analyzed for TAF content using HPLC. As human skin from different donors was employed for the different permeation studies, the in vitro permeation data was normalized using the flux that was obtained by repeating permeation of a previously evaluated patch, within every new permeation study. Permeation flux was calculated from the slope of the linear profile of amount of TAF permeated/ cm^2^ and time. The average of all the replicates ± SE has been reported. The calculated flux was extrapolated to patch size of 50 cm^2^ and time duration of 24 h, in order to calculate the dose administered/day.

#### 2.2.5. In Vitro Drug Release Studies

The release profile of TAF from the optimized patch (SP7) was evaluated using Paddle over Disk, USP V dissolution bath apparatus (Sotax AT7 Smart, Sotax AG, Switzerland) [26,27]. This study was performed during week 1 and after 1.5 and 3 months of patch preparation and storage at RT and 40 °C. The patches (0.712 cm^2^) were applied on the teflon mesh and then placed on the glass discs, such that the release surface was facing upwards (*n* = 3). The distance between the paddle and the surface of the disk was about 25 mm. The disks were placed at the bottom of the vessels containing 500 mL of phosphate buffer (pH 6) as the receptor media. Temperature of the media was set at 32 ± 0.5 °C and the paddle speed was 100 rpm. The samples (1 mL) were drawn at 0, 2, 4, 6, 8 h with replacement with fresh media and the entire receptor (500 mL) was removed and replaced with fresh buffer at 24, 48, 72, 96, 120, 144, 168 h. All the samples were analyzed for TAF content using HPLC. Results have been presented as average cumulative amount of TAF released ± SE. The average percentage of TAF released after 7 days from the tested patches has been reported as well and was calculated for each replicate using the following equation:

$$\%TAF\;released=\left(\frac{Cumulative\;amount\;of\;TAF\;released\;over\;7\;days}{Total\;amount\;of\;TAF\;in\;patch\;tested}\right)\;\times 100\%$$

#### 2.2.6. Coat Thickness, Coat Weight, and Drug Content of Optimized Patch

Coat thickness of the 1 cm^2^ laminates of SP7 (from different areas of the patch) was determined by measuring the patch thickness using a thickness gauge (Cedarhurst, NY, USA), and then subtracting the thickness of the release liner and backing membrane from the former. The coat weight and drug content of the optimized patch were determined using the procedure described in Section 2.2.3. These parameters were determined at day 1 and after 1.5 and 3 months of storage of patch at RT and 40 °C (*n* = 4–6).

#### 2.2.7. Quantitative Analysis

A UV detection based reverse phase HPLC method was used for quantitative analysis of TAF. Waters Alliance 2695 separation module (Milford, MA, USA) that was attached to a 2996 photodiode array detector was used. Isocratic elution was performed on Phenomenex Luna 5µ C8 (2) 100A, 250 × 4.6 mm (Phenomenex, CA, USA), at a flow rate of 1.0 mL/min. and column temperature of 35 °C, after injecting 50 µL of sample. The mobile phase consisted of acetonitrile (phase A) and 0.1% *v*/*v* trifluoroacetic acid in DI water (phase B) in the ratio of 30:70. The run time was 12 min. and the retention time of TAF was around 7.4 min. The drug standards were prepared in the receptor solution and were detected at wavelength of 262 nm. The precision limit of detection and quantification were 0.01 μg/mL and 0.03 μg/mL, respectively, and linearity was observed in the concentration range of 0.1–50 µg/mL (*R*^2^ = 0.9999).

#### 2.2.8. Physical Characterizations of Optimized Patch

##### Peel Adhesion

The 180° peel adhesion tester (ChemInstruments, Fairfield, OH, USA) was used to evaluate the peel adhesion force (force that is required to peel away the transdermal patch from dermatomed human cadaver skin) [28]. The instrument was calibrated for experimental parameters, such as tension, speed, and peel length with a load cell weighing 50 g prior to running the test patches. Transdermal patches with the length and width of 3.0 inches and 1.0 inch, respectively, were cut. One end of the adhesive coated backing membrane was adhered to the human skin affixed on the stainless steel plate and the other end was attached to load cell grip. The average force that is required to peel off the adhesive film was measured and recorded during the first week after patch preparation at RT and after three months of its storage at RT and 40 °C (*n* = 3) as well. The results have been shown as average ± SD.

##### Tack Properties

A texture analyzer (Texture Technologies Corp, Marietta, GA, USA) consisting of a 7 mm probe was used to determine the adhesion efficiency of the TAF suspension patch [29]. Prior to running the test samples, the instrument was calibrated and parameters, such as target force, approach speed, return speed, as well as distance and hold time, were optimized. A transdermal patch (width and length of about 1.0 inch × 1.0 inch, respectively) was cut and adhered on to the sample holder after removal of release liner. As the test run was initiated, the probe was allowed to touch the adhesive film with a target force and hold time of 50 g and 10 s, respectively. This resulted in the creation of a bond between the probe surface and transdermal patch. Further, as the probe was pulled off, it resulted in debonding between the two surfaces. Parameters, such as work of adhesion, positive area, and separation distance were hence recorded during the first week after patch preparation at RT and after three months of storage at RT and 40 °C (*n* = 3). The results have been shown as average ± SD.

#### 2.2.9. Evaluation of Skin Irritation Potential of Optimized Patch

An in vitro EpiDerm^TM^ skin irritation test (EPI-200-SIT) with a three-dimensional (3D) in vitro reconstructed epidermis (RhE) model (MatTek Corporation 200 Homer Ave, Ashland, MA 01721) was employed to assess the cell viability of skin while using a methyl thiazolyl tetrazolium viability assay [30] after the application of patch SP7. Three replicates of each of the patches were tested and compared to a negative control (Dulbecco’s phosphate buffered saline) and positive control (5% aqueous sodium dodecyl sulfate solution). After the removal of the release liners, patches were adhered to the tissues and kept in an incubator for 1 h at 37 °C. Afterwards, the patches were removed from the surface of tissue inserts, the inserts were washed using PBS, and then transferred to a fresh assay medium for 24 h incubation. The media for the inserts was exchanged and incubated again for 18 h. This was followed by transferring the inserts into yellow methyl thiazolyl tetrazolium solution and incubation for 3 h. During the 3 h incubation, mitochondrial metabolism was expected to occur and it was detected by the formation of a purple-blue formazan salt. The plate with the inserts was filled with isopropyl alcohol (2 mL) and kept on a shaker at 120 rpm for 2 -3 h. The aliquots were then transferred to a 96 well enzyme linked immunosorbent assay (ELISA) plate and the optical density of the extracted formazan salt was measured at 560 nm while using a Synergy HT plate reader (BioTek Instruments, Inc, Winooski, VT, USA).

#### 2.2.10. Data Analysis

Microsoft Excel and SPSS software package version 21.0 (IBM, USA) were used for analyzing data. Single factor analysis of variance (ANOVA) and Student’s *t*-test were used for statistical analysis and a *p* value of less than 0.05 was considered for concluding significant difference between the test groups.

## 3. Results and Discussion

### 3.1. Slide Crystallization Studies

Adhesiveness is a fundamental property of transdermal patches that is essentially required to ensure the complete contact between the entire surface area of the patch and skin during the wear period, for the efficient delivery of drugs. PSAs deform upon the application of slight pressure, provide intimate contact with surfaces by establishing inter-atomic and molecular forces at the interface, and are thus used for the preparation of transdermal patches [31]. A good adhesive is one that does not leave any residue upon removal, is easy to use, stable to environmental changes, non-irritant and non-sensitive to skin, compatible with other formulation components, allows sufficient drug solubility, and possesses the necessary adhesive properties, such as tack, shear, and skin adhesion [22,32]. Acrylic, silicone, and PIB-based adhesives are most commonly used in the design of transdermal patches [33]. Therefore, all three types of adhesives (acrylate: DURO-TAK 87-2516, silicone: BIO-PSA 7-4301, and PIB: DURO-TAK 87-6908) were explored for formulation development of TAF transdermal patches.

**Figure 1:**
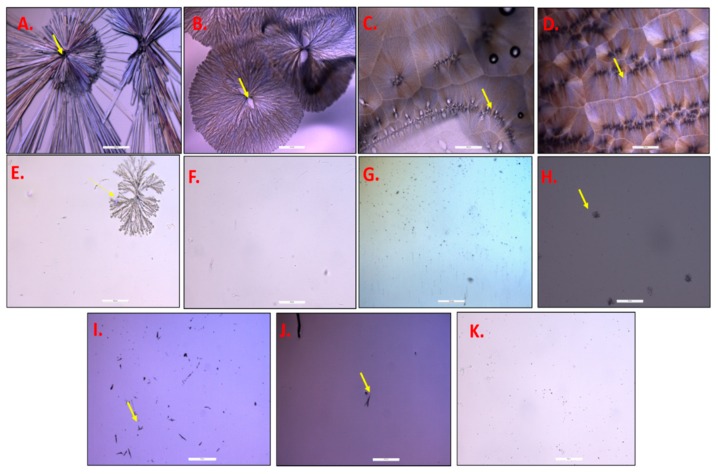
Images of slide crystallization studies, under optical microscope. (**A**). Pure TAF (at 10×). (**B**–**D**). TAF crystals in DURO-TAK 87-2516 immediately after evaporation of organic solvent at 10×: (**B**) 3% *w*/*w* TAF (**C**). 5% *w*/*w* TAF (**D**). 12% *w*/*w* TAF. (**E**). 2% *w*/*w* TAF in DURO-TAK 87-2516, 3 days after evaporation of organic solvent (10×). (**F**). 1% *w*/*w* TAF in DURO-TAK 87-2516, 7 days after evaporation of organic solvent (20×). (**G**). 4% *w*/*w* TAF in 5% *w*/*w* OA in DURO-TAK 87-2516 (10×). (**H**). 5% *w*/*w* TAF in 5% *w*/*w* OA in DURO-TAK 87-2516 (20×). (**I**). TAF:PVP 360 (9:1) under 20×. (**J**). TAF:PVP 360 (8:2) under 10×. (**K**).TAF:PVP 360 (7:3) under 20× (arrows depicting TAF crystals).

The determination of saturation solubility of TAF in different adhesives was required to determine the maximum amount of dissolvable drug that could be incorporated in the adhesives. Slide crystallization is a preliminary and relatively fast method that is employed as an alternate to preparing complete patches for estimating the solubility of drugs in adhesives [21]. Images of pure TAF, as observed under the microscope at a magnification of 10×, are shown in Figure 1A. TAF, at a concentration of 1% *w*/*w* (dry weight), was not soluble in silicone (BIO-PSA 7-4301) as well as PIB (DURO-TAK 87-6908) adhesive, even before the evaporation of solvents. Therefore, due to poor solubility in these adhesives, further slide crystallization studies were not performed with them. However, TAF blends at a concentration of 1–12% *w*/*w* (dry weight) in DURO-TAK 87-2516 were successfully prepared and drops of these were allowed to dry on glass slides and images of dried drug-adhesive blends, as observed under the microscope, are shown in Figure 1B–F. TAF at concentrations of 3–12% *w*/*w* was observed to immediately crystallize after drying (Figure 1B–D). However, 2% *w*/*w* TAF blend crystallized after three days (Figure 1E) and 1% *w*/*w* blend did not crystallize, even after seven days at RT (Figure 1F). Therefore, the saturation solubility of TAF in DURO-TAK 87-2516 was observed to be between 1–2% *w*/*w* (dry weight). Moreover, the addition of 5% *w*/*w* OA to DURO-TAK 87-2516 adhesive enhanced the solubility of TAF to about 4% *w*/*w*. The blend containing 5% *w*/*w* TAF in adhesive-OA blend showed crystals after seven days, whereas the 4% *w*/*w* TAF solution did not, as shown in Figure 1H,G, respectively. Further, in order to select the composition ratios of different crystallization inhibitors and TAF for patch formulations with 5%, 10%, and 15% *w*/*w* TAF (higher than its solubility in adhesive alone), different ratios of the two components were tested in slide crystallization studies. The mixtures of TAF and PVP 360 in the ratios of 9:1 and 8:2 showed the appearance of crystals after seven days, but the compositions with TAF:PVP ratios from 7:3 to 1:9 did not show any crystals (Figure 1I–K). All of the other crystallization inhibitors (Kollidon^®^ VA64, Kollidon^®^ 90F, and Kollidon^®^ 30LP) with TAF, each in the ratio of 1:9, did not show appearance of any crystals after seven days. Therefore, ratios of 7:3 (TAF:PVP 360) and 9:1 (TAF: Kollidon^®^ VA64 or Kollidon^®^ 90F or Kollidon^®^ 30LP), comprising of the lowest amount of PVP with the potential to inhibit crystallization, were selected for the preparation of patches containing 5% *w*/*w* or higher concentrations of TAF.

### 3.2. Formulation of TAF Acrylate Patches

Slide crystallization is a faster means for estimating the saturation solubility of drugs in adhesives. However, the thickness of the actual patches, as well as processing conditions and scale up procedures, may affect the phenomenon of crystallization. Therefore, based on the observations of slide crystallization studies, different transparent TAF patches were prepared in DURO-TAK 87-2516 adhesive, as shown in Table 1 and observed for crystallization for six months under the microscope. The solubility of TAF in 5% *w*/*w* OA in DURO-TAK 87-2516 blend (dry weight basis), as determined by slide crystallization studies, was found to be about 4%. Therefore, 2%, 3%, and 4% TAF patches (200 gsm) were prepared in this matrix (AP1, AP2, AP3, as specified in Table 1). Due to the solubility limitations of TAF in the adhesive, it was not possible to prepare patches with higher drug concentrations in the absence of crystallization inhibiting agents that act as solubilizers and anti-nucleants. The use of different grades of PVP as crystallization inhibitors has been well-reported in literature and some of them were, hence, investigated in the current study [22,34,35]. In order to prepare 100 gsm patches containing higher percentage of TAF (5%, 10%, and 15% *w*/*w*—AP4, AP5, AP6, respectively), PVP 360 was added as a solubilizer and crystallization inhibitor, with drug and PVP in the ratio of 7:3, in addition to OA. Patch ‘AP10′ was prepared to investigate the effect of enhancing the percentage of PVP 360, as well as the addition of 5% *w*/*w* PG in the 7.5% TAF patch on drug crystallization (100 gsm). Drug and PVP were in the ratio of 6:4 (*w*/*w*) in this formulation. Formulations AP7 to AP9 included the addition of other grades of PVP (Kollidon^®^ VA64, Kollidon^®^ 90F, and Kollidon^®^ 30LP) as solubilizers and crystallization inhibitors with TAF (10% *w*/*w*), in the ratio of 1:9 (PVP: TAF) in OA and DURO-TAK 87-2516 blend. Patch AP11 (100 gsm) was prepared to investigate the effect of enhancing the percentage of Kollidon^®^ 30LP as well as the addition of 5% *w*/*w* PG in the 7.5% TAF patch on drug crystallization. Drug and Kollidon^®^ 30LP were in the ratio of 7:3 (*w*/*w*) in this formulation. Further, patches AP12 to AP14 (400 gsm) containing 2% *w*/*w* TAF were prepared to evaluate the effect of combination of permeation enhancers (5% *w*/*w* OA+ 5% *w*/*w* PG + 5% *w*/*w* oleyl alcohol, 5% *w*/*w* OA+ 5% *w*/*w* PG + 5% *w*/*w* triacetin, 5% *w*/*w* OA+ 5% *w*/*w* PG + 8.5% *w*/*w* octisalate, respectively), as well as increasing the coat weight on the skin permeation of TAF and comparing it with that of AP1. Further, the AP15 (400 gsm) patch was prepared by replacing 5% *w*/*w* OA in AP12 with 5% oleyl alcohol, such that the total oleyl alcohol content was 10% *w*/*w* in this formulation. The transdermal permeation enhancing effects of OA [36,37,38], oleyl alcohol [39,40], triacetin [41,42,43], and octisalate [44,45] are well-known and they have been reported in literature, and thus these chemical enhancers were selected for the formulation development of TAF patches.

#### Microscopy and Stability Assessment of TAF Acrylate Patches

**Figure 2:**
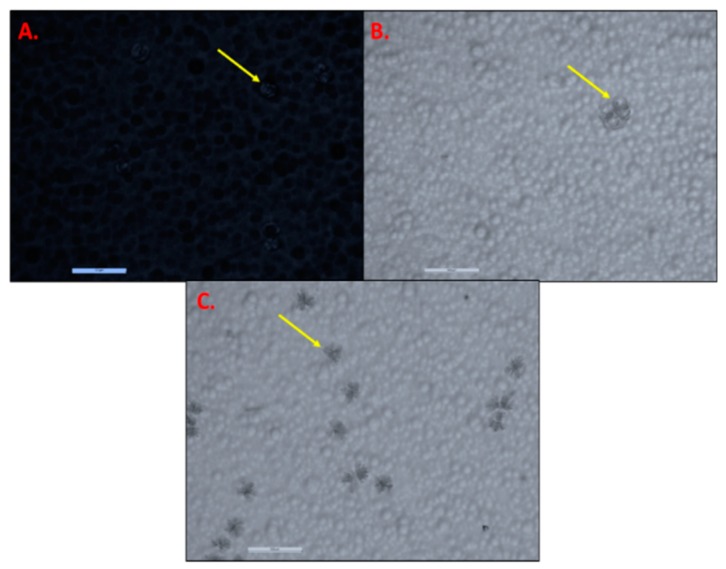
TAF crystals in acrylate based transdermal patches (at 10×). (**A**) Patch AP5 (crystals at 40 °C after 2 weeks). (**B**) Patch AP9 (crystals at RT in 12 days). (**C**) Patch AP8 (crystals at RT in first week). Arrows depict TAF crystals

During the storage period, changes in temperature can alter the thermodynamic activity of the transdermal patch formulation, and they result in precipitation or crystallization or changes in the crystal habit of the active ingredient. Therefore, during stability testing, patches are exposed to stress and real world storage conditions that are representative of the product’s proposed marketing conditions [46]. The stability of the acrylate based TAF patches was assessed in terms of the occurrence of crystallization over a period of time at different temperature conditions. Table 4 shows the microscopic observations of the same after six months. Figure 2 shows the representative images of TAF crystals in transdermal patches.

Patches with 2% *w*/*w* TAF were found to be stable and they did not show crystals at all of the tested temperature conditions, even after six months, as the drug concentrations were within the saturation solubility limits in these matrices. However, in order to incorporate drugs in amounts that are higher than its saturation solubility in the adhesive alone, crystallization inhibitors were included in the patches. Mechanistically, these additives stabilize the systems against crystallization, either due to enhancing the solubility of the drug or by aiding adsorption of drug crystals in the adhesive matrix. PVP has been previously demonstrated as one of the most effective additives in inhibiting drug crystallization in patch formulations acting by the formation of amorphous co-precipitates with the drugs [35,47,48,49]. Additionally, it has been previously shown that PVP inhibits the crystallization of drugs by being adsorbed on the growing crystal surfaces [35,50]. Therefore, different grades of PVP were incorporated in patches containing higher concentrations of TAF. However, patches with 15% *w*/*w* TAF (AP6) and PVP 360, crystallized in less than ten days at RT and 40 °C, depicting the inability of PVP 360 to inhibit crystallization at high drug levels. At lower drug concentrations (5% and 10% *w*/*w* TAF, AP4, and AP5, respectively), the addition of PVP 360 in the same ratio with TAF (3:7, *w*/*w*), as in AP6, slowed down the process of crystallization, where it was observed after three and two weeks, respectively, at RT as well as 40 °C. Further, increasing the PVP:TAF ratio to 4:6 (*w*/*w*) and adding PG as additional solubilizer in patch AP10 formulation did not delay the process of crystallization further and crystals were still observed in less than a month as well at RT and 40 °C. Interestingly, these patches did not show the presence of any crystals, even after six months at −20 ° C. Further, other crystallization inhibitors (Kollidon^®^ VA64, Kollidon^®^ 90F) were found to be ineffective, as crystals were visible during the first week after storage of all these patches at RT as well as 40 °C. However, the crystals that were observed in patch AP9 (containing Kollidon^®^ 30LP) were smaller than AP7 and AP8 and they were observed after about two weeks at RT and after first week at 40 °C. Therefore, patch AP11 was formulated with reduced drug concentration (7.5% *w*/*w* TAF), a higher amount of Kollidon^®^ 30LP than AP9, and the addition of PG, and showed a slower appearance of crystals (observed after three weeks at RT). However, the inability of PVP to prevent the appearance of TAF crystals in the transdermal patch formulation for longer time duration may be attributed to the difficulty in the molecular interactions between PVP and growing TAF crystals, because of the higher viscosity of the patch formulation as compared to a single drop of blend on the slides [35]. Overall, although most of the studies showed PVP to be an effective drug crystallization inhibitor, there are a few findings regarding ineffectiveness of some grades of PVP. For example, Weng et al. reported that PVP K30 was found to be ineffective in inhibiting the crystallization of risperidone in drug-in-acrylate adhesive transdermal patches. However, in the same study, OA was found to be an effective crystallization inhibitor [34]. In this study, all the compositions of 2% *w*/*w* TAF transdermal patches were found to be stable at all of the temperature conditions for more than six months, which may be attributed to drug solubility in the adhesive matrix being lower than the saturation level, as well as the crystallization inhibiting effect that is conferred by OA. Additionally, the transdermal patches formulated with higher drug concentrations (AP2, AP3, AP4, AP5, AP10, AP11) were only stable at −20 °C.

### 3.3. Formulation of TAF Suspension Patches

Due to low solubility of TAF in silicone and PIB adhesives, the drug was suspended in the blends of these adhesives with different additives, as elaborated in Table 2. Mineral oil was added as a plasticizer that increases diffusivity of the drug by decreasing the resistance that is offered by the patch matrix [22]. In addition to the plasticizing effect, mineral oil has also been reported as a transdermal permeation enhancer [51]. OA and oleyl alcohol are also well-known chemical penetration enhancers [36,37,38,39,40] that were included in the TAF suspension patches. A number of different silicone based TAF patches were prepared in order to select the optimal type of the release liner and the backing membrane. Homogenization speed and time were also optimized to reduce the particle size of the drug.

Patch SP1 (~100 gsm) was prepared while using the fluoropolymer coated side of Scotchpak^TM^ 1022 and Scotchpak^TM^ 9733 (polyester backing). For TAF suspension patch formulations, SP2-SP13, a higher coat weight (~200–350 gsm) was applied and oleyl alcohol was included as an additional enhancer. For the preparation of SP2 patch, the same release liner and backing membrane as SP1 were initially selected. However, in terms of the TAF suspension patch formulation with oleyl alcohol, it was found that, when the formulation was coated on the fluoropolymer coated side of the liner, it could not come off the release liner, depicting the affinity of the formulation binding to the fluoropolymer coated release liner was more than the polyester backing membrane. Therefore, the formulation was casted on the uncoated side of the release liner (polyester only) and then laminated while using the polyester (Scotchpak^TM^ 9733) backing film. This was an interesting experimental observation, as silicone based transdermal patches are usually casted on the fluoropolymer coated side of release liner in order to have easy release/transfer of the formulation from the liner to backing. Further, when the patch formulation SP2 was peeled off, it was observed that the adhesive layer transiently formed a film on the skin, depicting less affinity of the formulation for polyester backing material.

Therefore, other materials, such as EVAC based (Corona-treated - CoTran™ 9702, 9706, 9728) and polyethylene based (CoTran™ 9720 and CoTran™ 9718), were tried for the optimization of the backing membrane, while the uncoated side of Scotchpak^TM^ 1022 was used as the release liner for the patch formulations, SP3-SP6. The formulation SP5 and SP6, using CoTran™ 9718 as the backing membrane, showed comparatively better peeling characteristics than the SP4 patch. The SP3 patch could not be successfully prepared, as the formulation film did not come off from the liner onto the EVAC backing membranes. Higher speed and duration of homogenization was employed for formulation SP6 when compared to that SP5 in an attempt to investigate the effect of homogenization with different parameters on the drug particle size and skin permeation. However, the issue of the residual film of the formulation on skin was not completely resolved in SP5 and SP6, indicating the requirement of a more compatible backing membrane material to facilitate the peeling off process of the patch formulation. In addition, upon long term storage (after a month), patches SP5 and SP6 showed the contraction of the film and the appearance of streaks.

As was evident from patches SP2-SP6, the silicone-based TAF suspension patch formulation did not have sufficient affinity to the backing membrane materials, including polyester and polyethylene. Therefore, silicone coated PET films (48101-4400B/000, 44916-7300AM/000, and or 40987-U4162/000) were explored as the backing membrane for SP7, SP8, and SP9, respectively. Patch SP7 (containing 15% *w*/*w* TAF) showed the best efficiency in terms of least resistance and minimal formulation residue, while peeling the patch formulation off from the skin. Hence, patches SP10-13 were prepared while using the same material components and processing parameters as SP7, except that different concentrations of TAF (5, 10, 20, 25% *w*/*w*) were included in these patches. For the preparation of PIB-based TAF suspension patch formulation (SP14), Scotchpak^TM^ 1022 (Fluoropolymer coated side) and Scotchpak^TM^ 9733 were used as the release liner and the backing membrane, respectively.

#### 3.3.1. Visual Observation of TAF Suspension Patches

Table 5 summarizes the details of the visual observations of the different TAF suspension patches. Patches SP1, SP7, SP10-13, and SP14 were observed to be acceptable in terms of the properties that are specified in the table. The visual observations depicted that the addition of oleyl alcohol in the silicone suspension formulation rendered it more lipophilic, and thus provided more affinity or binding with skin. Using a silicone-coated film as a backing membrane resolved the issue of the residual film after peeling off. The PIB patch formulation (SP14) did not have any issues during the peeling off process as compared to the silicone patch formulations.

#### 3.3.2. Effect of Homogenization on the Particle Size of TAF

The effect of homogenization parameters on the particle size of TAF was also investigated, in order to eventually evaluate the effect of the particle size of API in the adhesive matrix on the skin permeation. Homogenization is a process that consists of micronizing or reducing the particle size of dispersions by the application of high sheer, pressure, turbulence, as well as acceleration and impact [52]. Decreased particle size would aid in enhancing the solubility of the drug in the adhesive formulation, and therefore improving its permeation rate [53]. The effect of homogenization speed and duration on the particle size of API has been previously reported, and thus similar parameters were selected to reduce the particle size of TAF [54,55]. As reported, homogenization speed is an indicator of the amount of energy that is applied to the system, as determined by the velocity of the rotating mixing heads. Further, the mechanical impingement of the particles against the wall, due to the high acceleration of the fluid and shear stress in the gap between the rotor and stator, leads to the reduction in the particle size of the drug substance [54].

**Figure 3:**
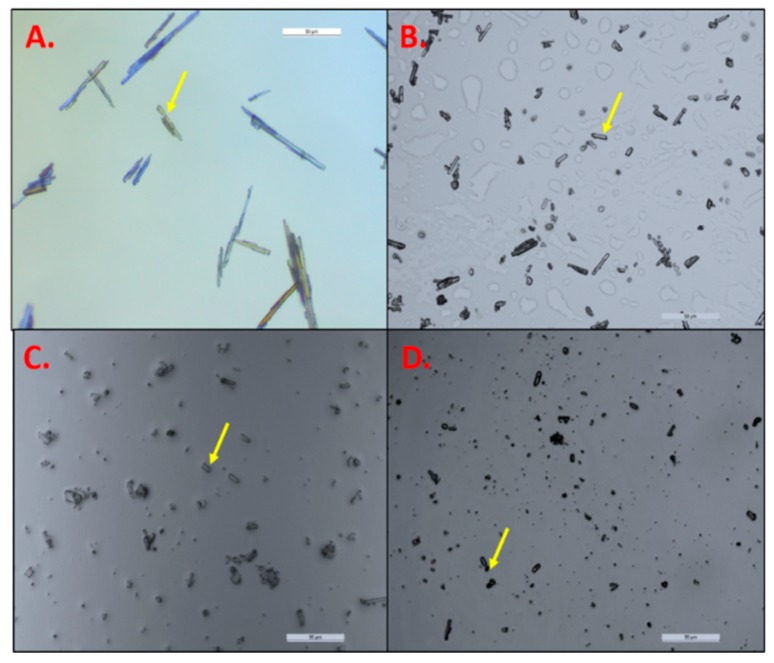
(**A**) Microscopic images of pure TAF particles suspended in heptane; TAF particles in suspension formulation, before addition of adhesive (at 20×) after homogenization at: (**B**) 30,000 rpm for 20 min; (**C**) 32,000 rpm for 30 min; (**D**) 32,000 rpm for 5 min.

The average particle size of the pure TAF was observed to be 53.39 ± 16.15 µm under the optical microscope (Figure 3A). After homogenization at 30,000 rpm for 20 min, the particle size of TAF was reduced to 11.51 ± 2.89 µm (*p* < 0.05) (Figure 3B). Further increasing the speed to 32,000 rpm and the homogenization time to 30 min. significantly reduced the particle size of TAF to 6.0 ± 1.8 µm as compared to that of homogenization at 30,000 rpm for 20 min. (*p* < 0.05) (Figure 3C). The observation of the reduction in the particle size of API after the increase in the speed and time of homogenization was in concordance with those that were reported previously [54]. As shown in Figure 3C, some of the particles were smaller and were not visible under the microscope, and thus could not be measured using the software. However, it was found that homogenization at 32,000 rpm over 5 min. resulted in a similar reduction in drug particle size (5.28 ± 2.71 µm, Figure 3D) to that of homogenization at 32,000 rpm for 30 min. Collectively, the speed of homogenization had relatively more impact on the reduction in particle size as compared to the duration of the homogenization. Thus, 32,000 rpm and 5 min. were the homogenization parameters that were employed for the formulation of SP7-SP13.

### 3.4. Coat Weight and Drug Content

Coat weight and drug content of the patches evaluated for the in vitro permeation studies were measured and are reported in Table 3. These measured properties are influenced by various factors, such as percentage of non-volatile components in the adhesive blend, scale set-up, surface level of the casting knife, textural properties, of release liner and backing membrane, and they are reflective of the coating efficiency of the patches. The results showed that all of the patches (acrylate, silicone, PIB) had uniformity in both coat weight and drug assay. Additionally, the values of theoretical and experimental drug content were close for all of the patches that confirmed the stability of the drug after exposure to the temperature conditions used for the processing of respective patches.

### 3.5. In Vitro Permeation Studies

#### 3.5.1. Epidermal Integrity and Thickness Assessment

The integrity of human epidermis samples must be evaluated prior to permeation studies, as the procedures that are employed for procurement of human skin, such as surgical removal, dermatoming, and storage, as well as technical procedure for the separation of epidermis from dermatomed skin, can damage the skin, and thus ultimately influence drug permeation. The physical integrity of stratum corneum has been reported to be indicated by its electrical properties. Therefore, the measurement of electrical conductivity is used as a means of assessing the barrier integrity for full thickness skin as well as epidermal membranes [27,56]. Hence, for the selection of epidermal pieces with optimum barrier integrity, electrical resistance of skin was measured and considered to be the main selection criterion. Epidermal pieces with the resistance above 10 kΩ, were selected for the study. Further, the thickness of human epidermis used for the permeation studies ranged from 50–150 µm.

#### 3.5.2. Selection of Receptor Solution

Table 6 shows the percentage degradation of TAF in different receptor solutions at 37 °C. It was evident that TAF degrades in PBS, pH 7.4, with or without PEG 400. Lowering the pH to 6.0 and using non-aqueous media, such as pure PEG 400, reduced the degradation of TAF. However, due to limitations, such as high viscosity as well as low drug sensitivity in HPLC, PEG 400 was not selected as the receptor media. Phosphate buffer, pH 6.0 was thus selected as the receptor solution, and it was completely replaced every 24 h during the seven-day skin permeation studies. The degradation of TAF at both acidic and basic pH has been previously reported in the literature. Additionally, TAF has been found to be stable at pH of around 5–6.8 [57]. In addition, the solubility of TAF in PBS was found to be 4.95 ± 0.07 mg/mL. Experimental conditions ensured the maintenance of sink conditions for our in vitro permeation studies.

#### 3.5.3. Determination of Permeation Flux of TAF Transdermal Patches

Table 3 describes the different patches that were evaluated in seven day in vitro permeation studies.

##### Permeation of TAF from Acrylate-Based Clear Patches: Effect of Drug Concentration

**Figure 4:**
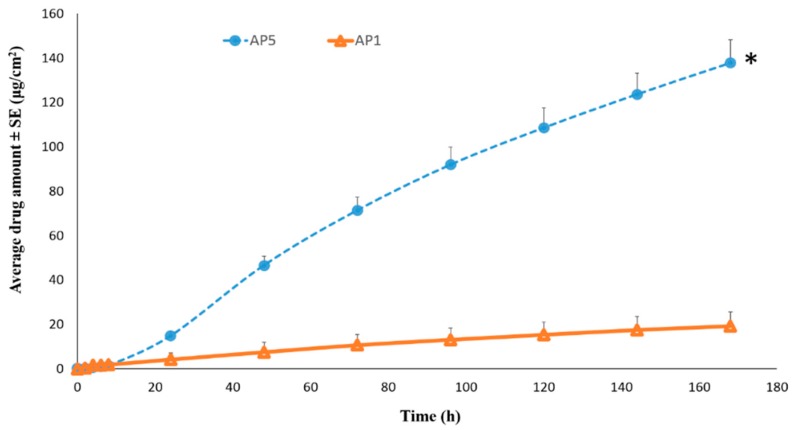
Permeation profiles of TAF from acrylate based patches across human epidermis. * Represents statistical significant difference, Student’s t test (*p* < 0.05). AP1: Acrylate patch containing 2% TAF, AP5: Acrylate patch containing 10% TAF.

The amount of TAF that permeated from patch AP1 (simplest matrix comprising of 2% *w*/*w* TAF dissolved in mixture of OA in acrylate adhesive) after seven days was found to be 19.20 ± 2.92 µg/cm^2^. The average permeation flux over duration of seven days was calculated to be 0.12 ± 0.01 µg/cm^2^/h. To evaluate the effect of drug concentration in the patch on its skin permeation, patch AP5 with 10% *w*/*w* TAF, which was found to be stable for about two weeks at RT, was selected for an in vitro permeation study. As shown in Figure 4, increasing the concentration of TAF to 10% *w*/*w* (AP5) significantly enhanced the drug permeation to 137.76 ± 10.42 µg/cm^2^ after seven days and flux rate was calculated to be 0.88 ± 0.07 µg/cm^2^/h as compared to that of AP1 (*p* < 0.05). Thus, increasing the drug concentration significantly enhanced its permeation across human epidermis. However, since AP5, as well as other acrylate adhesive-based patches with a TAF concentration of more than 2% (*w*/*w*), crystallized at RT over a period of time, transdermal patches with 2% *w*/*w* TAF, and consisting of combination of chemical penetration enhancers as well as higher coat weight, were prepared and evaluated for skin permeation testing (AP12 to AP15).

##### Permeation of TAF from Acrylate-Based Clear Patches: Effect of Coat Weight and Chemical Penetration Enhancers

**Figure 5:**
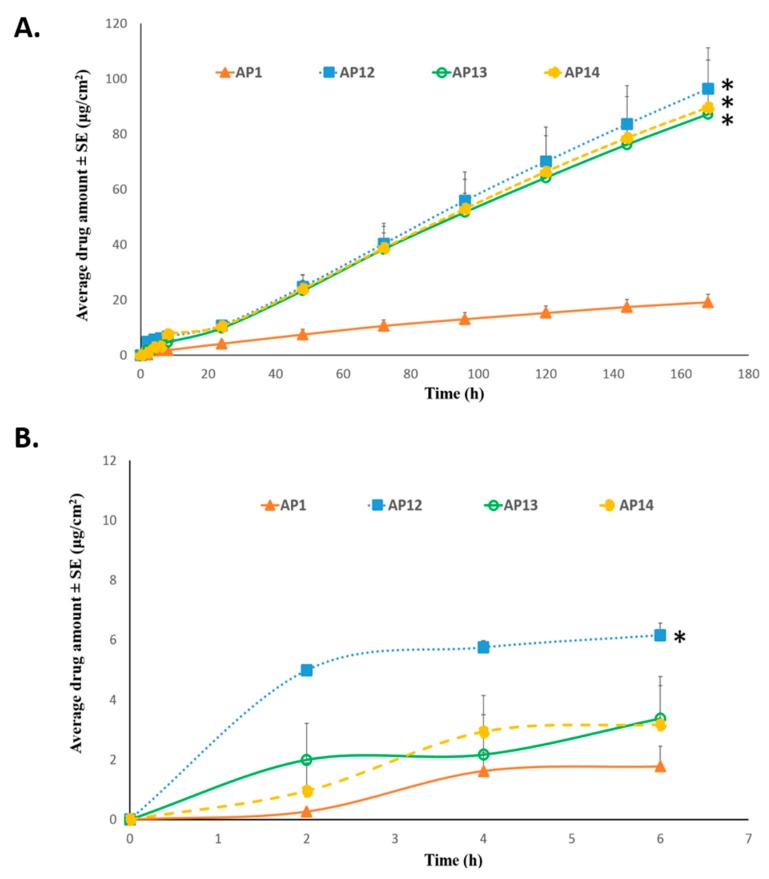
Effect of combination of enhancers and higher patch coat weight on permeation of TAF across human epidermis from acrylate patches: (**A**) Seven day permeation profile (**B**) Six hours permeation profile. * Represents statistically significant difference, ANOVA one way test (*p* < 0.05). AP1: Acrylate based patch containing 2% TAF and 5% oleic acid. AP12: Acrylate based patch containing 2% TAF, 5% oleic acid, 5% PG, and 5% oleyl alcohol. AP13: Acrylate based patch containing 2% TAF, 5% oleic acid, 5% PG, and 5% triacetin. AP14: Acrylate based patch containing 2% TAF, 5% oleic acid, 5% PG, and 8.5% octisalate.

**Figure 6:**
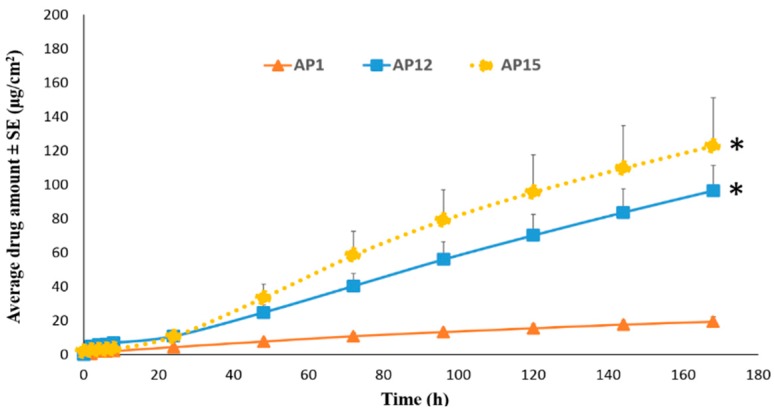
Permeation profile of acrylate based TAF patches comprising of oleyl alcohol and comparison with the control patch through human epidermis. * Represents statistically significant difference, ANOVA one way test (*p* < 0.05). AP1: Acrylate based patch containing 2% TAF and 5% oleic acid. AP12: Acrylate based patch containing 2% TAF, 5% oleic acid, 5% PG, and 5% oleyl alcohol. AP15: Acrylate based patch containing 2% TAF, 5% PG, and 10% oleyl alcohol.

As shown in Figure 5A, the cumulative amount of TAF permeation after seven days in the patches AP12 (96.40 ± 14.83 µg/cm^2^), AP13 (87.15 ± 8.60 µg/cm^2^), and AP14 (89.56 ± 17.28 µg/cm^2^) was found to be significantly higher than that of AP1 (19.20 ± 2.92 µg/cm^2^, *p* < 0.05). Additionally, the permeation flux that was observed from the three aforementioned groups was 0.60 ± 0.10 µg/cm^2^/h, 0.54 ± 0.05 µg/cm^2^/h, and 0.56 ± 0.12 µg/cm^2^/h, respectively, which was about five-fold greater than that observed from AP1 (0.12 ± 0.01 µg/cm^2^/h). Thus, increasing the patch coat weight to 400 gsm from 200 gsm along with the incorporation of a combination of chemical enhancers (OA+ PG+ oleyl alcohol, OA+ PG+ triacetin, or OA+ PG+ octisalate) resulted in the significant enhancement in drug permeation. However, no significant difference in TAF permeation between AP12, AP13, and AP14 was observed. As shown in Figure 5B, the amount of drug permeation over 6 h was significantly higher in patch AP12 (6.15 ± 0.41 µg/cm^2^) than AP13 (3.38 ± 1.40 µg/cm^2^), as well as AP14 (3.17 ± 1.30 µg/cm^2^, *p* < 0.05). Patch AP15 was formulated by replacing 5% *w*/*w* OA in AP12 with oleyl alcohol, thus having total of 10% *w*/*w* oleyl alcohol and a coat weight of 400 gsm. However, as shown in Figure 6, there was no significant difference between the amount of TAF permeation after seven days between the two groups (AP12: 96.40 ± 14.83 µg/cm^2^ and AP15: 122.69±28.34 µg/cm^2^, *p* > 0.05).

The effects of various chemical enhancers that were used in the patches, such as OA [36,37,38], oleyl alcohol [39,40], triacetin [41,42,43], and octisalate [44,45] on skin permeation enhancement are well-known. Synergistic permeation enhancing effect of the combination of PG and OA has also been reported earlier [25,39,58]. In our study, PG is expected to act as a cosolvent, and thus enhance the concentrations of the drug substance as well as the enhancer in the stratum corneum. OA, on the other hand, has the potential to delipidize the stratum corneum, and therefore facilitate the partitioning of the drug molecules and PG into skin [38,59]. Additionally, the synergistic effect of PG and oleyl alcohol was found to markedly increase the skin permeation of tenoxicam across hairless mouse skin [60]. Furthermore, a combination use of PG and oleyl alcohol, under occlusive conditions, has been reported to result in considerable enhancement in the permeation of testosterone across full thickness neonatal porcine skin. The mechanism of penetration enhancement by octisalate is not fully understood. However, due to the structural similarity, as well as similar observations in differential scanning calorimetry and attenuated total reflectance spectroscopy, in terms of the effect of delipidization on stratum corneum, octisalate is assumed to mechanistically work in the similar way to azone [44]. Additionally, octisalate has been incorporated as a penetration enhancer in sunscreens, cosmetic products, as well as in metered spray transdermal systems [45]. Further, triacetin has been incorporated as a penetration enhancer in the commercially available Oxytrol^®^ patch for overactive bladder [43,61]. Patel et al. also reported an enhancement in diclofenac permeation through human cadaver skin while using 10% triacetin [41].

##### Permeation of TAF from Suspension-Type Patches: PIB vs Silicone-Based

**Figure 7:**
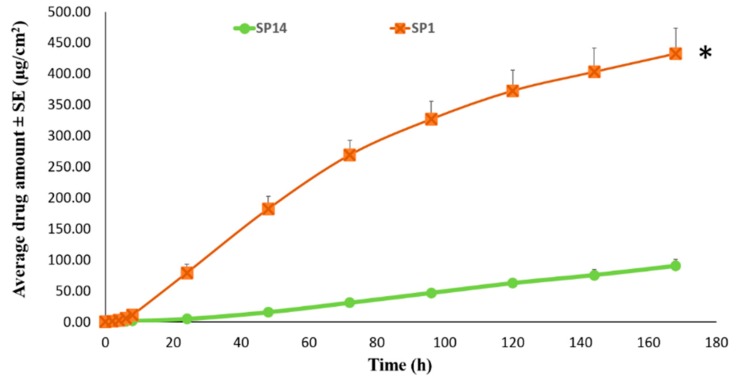
Comparison of permeation profile of silicone and PIB based TAF patches through human epidermis. * Represents statistical significant difference, Student’s t test (*p* < 0.05). SP1: Silicone based 15% TAF patch. SP14: Poly isobutylene based 15% TAF patch.

The PIB based TAF suspension patch (SP14) showed a permeation of 90.40 ± 10.18 µg/cm^2^ after seven days through human epidermis. A TAF permeation flux rate of 0.60 ± 0.07 µg/cm^2^/h and a lag time of about 19 h was observed with this patch. The results showed that the flux rate from SP14 was significantly higher than AP1 (*p* < 0.05), but was similar to that of AP12, AP13, AP14, and AP15 (*p* > 0.05). Further, silicone patch (SP1) with the same composition as PIB (SP14) was prepared. The cumulative amount of TAF that permeated from SP1 across human epidermis in seven days was observed to be 432.21 ± 41.25 µg/cm^2^ and it was significantly higher than all of the other patches evaluated previously (*p* < 0.05). Figure 7 shows the skin permeation profiles of PIB (SP14) and silicone (SP1) based patch. The resultant permeation flux from SP1 for seven days was found to be 3.39 ± 0.03 µg/cm^2^/h and the lag time was observed to be only 1.2 h. Further, with the addition of 10% *w*/*w* oleyl alcohol, as well as increased coat weight of ~250 gsm in patch SP5, the permeation flux of TAF was observed to significantly increase to 5.97 ± 0.47 µg/cm^2^ /h (*p* < 0.05). The total amount of TAF observed in the receptor after seven days was 958.07 ± 82.81 µg/cm^2^ and the lag time was about 1 h.

##### Permeation of TAF from Silicone-Based Suspension-Type Patches: Effect of Homogenization

**Figure 8:**
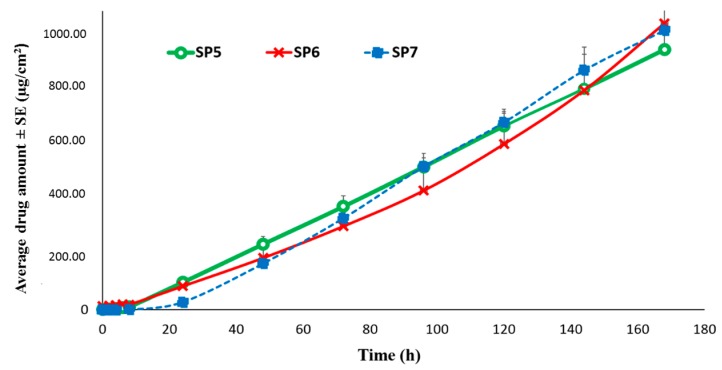
Comparison of permeation profile of silicone based TAF patches through human epidermis. SP5: Silicone-based 15% TAF patch prepared from formulation blend homogenized at 30,000 rpm for 20 min. SP6: Silicone-based 15% TAF patch prepared from formulation blend homogenized at 32,000 rpm for 30 min. SP7: Silicone-based 15% TAF patch prepared from formulation blend homogenized at 32,000 rpm for 5 min.

Briefly, the suspension blend for patch SP5 was homogenized at 30,000 rpm for 20 min, while homogenization at 32,000 rpm for 30 min. was performed for the preparation of SP6, to investigate the effect of homogenization parameters on the skin permeation. However, the amount of TAF permeation after seven days was not found to be significantly different between the two patch formulations (SP5: 958.07 ± 82.81 µg/cm^2^ and SP6: 1,054.38 ± 214.40 µg/cm^2^). It was found that a permeation flux rate of TAF from patch SP6 was 6.60 ± 1.48 µg/cm^2^/h and lag time of about 1 h was observed. Furthermore, the patch SP7 blend was prepared by homogenization at 32,000 rpm for only 5 min., and it did not show any significant difference in the amount of TAF permeation as well as the lag time when compared to those of SP5 and SP6 (*p* > 0.05), as shown in Figure 8. The permeation flux of TAF observed from patch SP7 was 7.24 ± 0.47 μg/cm^2^/h. Additionally, it can be inferred that the use of different backing materials in patches SP5/6 and SP7 did not impact the drug permeation across human epidermis.

##### Permeation of TAF from Silicone-Based Suspension-Type Patches: Effect of Drug Concentration

**Figure 9:**
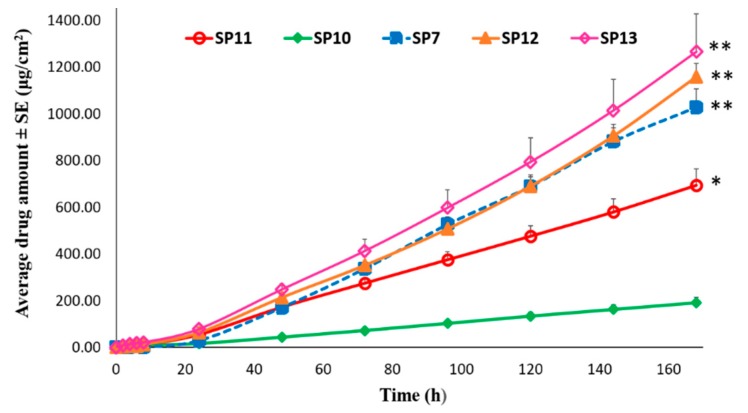
Effect of concentration of TAF in the silicone-based suspension patches on its permeation through human epidermis. * represents significant difference between 5 and 10% patch. ** represents significant difference as compared to 5 and 10% patch, ANOVA one-way test (*p* < 0.05). SP7: Silicone-based 15% TAF patch. SP10: Silicone-based 5% TAF patch. SP11: Silicone-based 10% TAF patch. SP12: Silicone-based 20% TAF patch. SP13: Silicone-based 25% TAF patch.

Silicone based suspension patches, SP10-13, were prepared to investigate the effect of concentration of TAF on its permeation profile across human epidermis. As shown in Figure 9, the cumulative amount of TAF permeated after seven days was found to be 191.51 ± 23.26, 695.09 ± 68.85, 1,028.69 ± 78.63, 1,158.86 ± 57.96, and 1,266.19 ± 162.04 µg/cm^2^ from SP10, SP11, SP7, SP12, and SP13, respectively. The permeation flux values for each of these patches were: 1.24 ± 0.13, 4.32 ± 0.47, 7.23 ± 0.58, 7.82 ± 0.48, and 8.44 ± 1.16 µg/cm^2^/h. A significant increase in permeation was observed with the increase in TAF concentration from 5 (SP10) to 10% *w*/*w* (SP11), as well as from 10 (SP11) to 15% *w*/*w* (SP7), (*p* < 0.05). However, there was no statistically significant difference between the amount of TAF that permeated from patches containing 15 (SP7), 20 (SP12), and 25% *w*/*w* (SP13) drug (*p* > 0.05). Thus, increasing the TAF concentration from 5 to 15% *w*/*w* resulted in a higher amount of undissolved drug as a reservoir in the latter that probably provided an additional and constant driving force, eventually aiding in considerably enhancing the permeation of TAF. However, increasing the drug concentration from 15 to 25% *w*/*w* did not result in any further enhancement in permeation. Therefore, 15% drug concentration (SP7) was found to be optimum in order to achieve the targeted permeation flux.

##### Permeation of TAF from Acrylate-Based Clear vs Silicone-Based Suspension-Type Patches

The aim of this study was to demonstrate a proof of concept for feasibility of developing a transdermal patch of TAF and achieve a clinically relevant flux of about 7 µg/cm^2^/h across human epidermis. From an in vivo perspective, as blood vessels are present in the dermis layer, once the drug crosses the stratum corneum and the viable epidermis, it reaches the systemic circulation. Hence, human epidermis was used in this study for the in vitro permeation testing. Owing to the desirable physicochemical properties (molecular weight= 476.47 g/mol, log P= 1.8), TAF showed permeation from the simplest transdermal patch formulation (AP1), across the human epidermis, depicting its potential to passively permeate through human skin. However, for the acrylate-based TAF patch formulations, it was only possible to achieve a flux rate of 0.6 µg/cm^2^/h, with a combination of different chemical penetration enhancers at a low drug concentration (2% *w*/*w*), with no issues regarding drug crystallization. However, the silicone based TAF suspension patch formulations containing 15–25% *w*/*w* drug showed higher permeation flux of about 7 µg/cm^2^/h, as well as a lower lag time (about 1h), which may be attributed to the release characteristics of TAF from the silicone adhesive matrix, and a higher drug loading in a suspension form, which can provide constant concentration gradient/driving force for the drug release and continuous permeation through epidermis. In addition, all of the excipients that were included in the TAF suspension patch—mineral oil, OA, and oleyl alcohol—have been previously reported for transdermal permeation enhancing effects, which may contribute to higher flux.

In our study, it was possible to achieve a relatively high flux rate of 7 µg/cm^2^/h, from silicone based TAF suspensions, and the optimized patch “SP7” was characterized for drug release, coat weight and thickness, and drug content after storage at RT and 40 °C for three months. This prototype patch was further characterized for physical properties, such as tack, shear, and peel adhesion.

### 3.6. In Vitro Drug Release Studies

**Figure 10:**
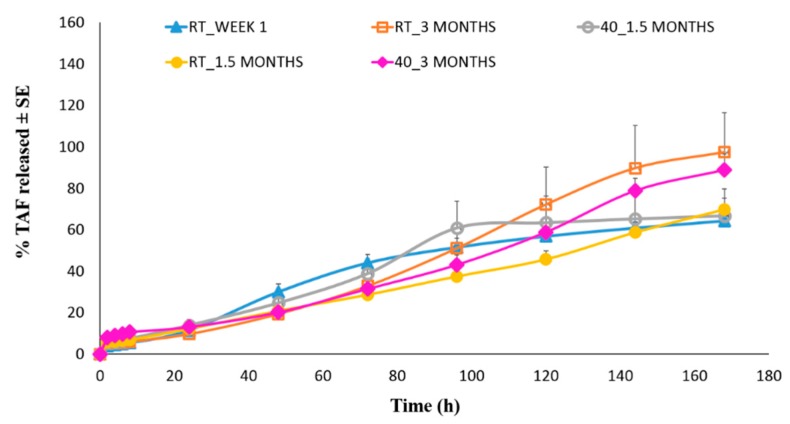
In vitro release profiles of TAF from the optimized suspension patch.

Figure 10 presents the seven-day in vitro release profiles of TAF from the optimized patch “SP7”. The average percentage of TAF released after 7 days was observed to be 64.27 ± 5.47, in the study performed during week 1 after the preparation of the patch. The release profile of TAF from the same batch of suspension patch was also studied after 1.5 and 3 months of storage at RT and 40 °C. Table 7 shows the results. No significant difference in the total percentage of TAF released from the patches that are exposed to different test conditions was observed (p>0.05). This depicted uniformity in the release profile and percentage of drug released after exposure to higher temperature for three months, further indicating the stability of TAF in the suspension-based patches.

### 3.7. Coat Thickness, Coat Weight, and Drug Content of Optimized Patch

Table 7 presents the average patch thickness, coat weight, and drug content of patch SP7, as measured after exposure to different temperature conditions for different durations. No significant difference in the measured parameters was observed after exposure of the patches to different test conditions (*p* > 0.05), signifying the stability and integrity of the suspension-based patches over time. Furthermore, as depicted by the results of drug content analysis, no degradation of TAF was observed after 1.5 and 3 months of patch storage at RT as well as 40 °C, further indicating the stability of TAF in the silicone-based suspension patches.

### 3.8. Physical Characterization of the Optimized Patch

#### 3.8.1. Peel Adhesion

An ideal transdermal patch should peel off after application on skin, without causing delamination. Peel adhesion is not only affected by the intrinsic adhesiveness of the PSA, but it also involves the stretching and the bending of the patch matrix, and also the backing layer prior to the separation. The force that is required to peel the patch should be consistent for different batches and the value of peel adhesion obtained in the test varies the width of the test material [62]. No significant difference (*p* > 0.05) in the average force that is required to peel the patch from human dermatomed skin was observed when tested during week 1 (202.78 ± 43.07 g) and three months after patch storage at RT (149.35 ± 30.76 g) and 40 °C (171.17 ± 13.56 g). No delamination was observed for the tested patches.

#### 3.8.2. Tack Properties

The adhesion efficiency of a transdermal patch can be tested by evaluating its tack, which is a measure of the force of debonding upon the application of a light pressure for a short time. A probe tack test was employed in this study, where the force that is required to separate a probe from the adhesive surface of a transdermal patch was measured. Tack was then expressed as the maximum value of the force that is required to break the bond between the probe and transdermal patch after a brief period of contact [62]. The average absolute positive force (adhesive or sticky property), average positive area (work of adhesion), and average separation distance (degree of legging), recorded for three replicates during the first week after patch preparation, was found to be 1,116.88 ± 167.79 g/cm^2^, 34.30 ± 10.98 g.s, and 1.27 ± 0.50 mm, respectively. Storage at RT (1,012.03 ± 220.59 g/cm^2^, 24.77 ± 8.50 g.s, and 0.8 ± 0.1 mm, respectively) and 40 °C (983.72 ± 201.56 g/cm^2^, 23.33 ± 6.20 g.s, and 1.0 ± 0.17 mm, respectively) for three months did not impact the tack properties of the optimized patch formulation (*p* > 0.05). Additionally, the average absolute positive force that was observed in our study was comparable to that observed for the 9.5% fentanyl containing silicone-based suspension patch laminate (1,499 g/cm^2^) reported in literature [63].

### 3.9. Evaluation of Skin Irritation Potential of Optimized Patch

The principle of irritation assay using the in vitro skin model is based on the premise that irritant chemicals can penetrate the stratum corneum by diffusion and they are cytotoxic to the cells in the underlying layers. In a transdermal patch, the drug by itself or in combination with other additives can be irritant to skin [30]. Hence, the entire patch was tested for its irritation potential, and not just the drug by itself. The tested transdermal patch (SP7) resulted in a mean relative cell viability of 104.64 ± 7.42 %, which was comparable to the negative control (100.00 ± 6.09) and significantly higher than the positive control (14.27 ± 8.09). Hence, it can be concluded that the final optimized silicone suspension patch (SP7) is non-irritant to human skin.

## 4. Conclusions

Several solution and suspension-type patch formulations were developed for the transdermal delivery of TAF. Ultimately, the optimized silicone-based suspension patch design successfully achieved the target release and duration profile for TAF and it was found to be suitable for weekly drug dosing. Drug permeation flux of 7 µg/cm^2^/h, as observed from the optimized patch, when extrapolated to 50 cm^2^ patch size, indicates the delivery of 8.4 mg TAF/day, surpassing the target dose of 8 mg/day. Further studies are underway to characterize its safety and pharmacokinetic profiles in vivo.

## Figures and Tables

**Table 1:** Composition of acrylate based Tenofovir alafenamide fumarate (TAF) transdermal patches.

Properties	Patch Formulation Codes														
	AP1	AP2	AP3	AP4	AP5	AP6	AP7	AP8	AP9	AP10	AP11	AP12	AP13	AP14	AP15
Coat weight (gsm)	200	200	200	100	100	100	100	100	100	100	100	400	400	400	400
**Composition**	**Components (% Dry Weight, *w*/*w*)**														
TAF	2	3	4	5	10	15	10	10	10	7.5	7.5	2	2	2	2
OA	5	5	5	5	5	5	5	5	5	5	5	5	5	5	-
DURO-TAK 87-2516	93	92	91	87.86	80.71	73.57	83.89	83.89	83.89	77.50	79.29	83	83	79.5	83
PVP 360	-	-	-	2.14	4.29	6.43	-	-	-	5	-	-	-	-	-
Kollidon^®^ VA64	-	-	-	-	-	-	1.11	-	-	-	-	-	-	-	-
Kollidon^®^ 90 F	-	-	-	-	-	-	-	1.11	-	-	-	-	-	-	-
Kollidon^®^ 30 LP	-	-	-	-	-	-	-	-	1.11	-	3.21	-	-	-	-
Oleyl alcohol	-	-	-	-	-	-	-	-	-	-	-	5	-	-	10
PG	-	-	-	-	-	-	-	-	-	5	5	5	5	5	5
Triacetin	-	-	-	-	-	-	-	-	-	-	-	-	5	-	-
Octisalate	-	-	-	-	-	-	-	-	-	-	-	-	-	8.5	-

**Table 2:** Composition and manufacturing parameters of the silicone based TAF suspension transdermal patches.

Properties	Patch Formulation Codes													
	SP1	SP2	SP3	SP4	SP5	SP6	SP7	SP8	SP9	SP10	SP11	SP12	SP13	SP14
Composition of matrix	Components (% Dry Weight, *w*/*w*)													
TAF	15	15	15	15	15	15	15	15	15	5	10	20	25	15
OA	5	5	5	5	5	5	5	5	5	5	5	5	5	5
Oleyl alcohol	-	10	10	10	10	10	10	10	10	10	10	10	10	-
Mineral oil	14	14	14	14	14	14	14	14	14	14	14	14	14	14
BIO-PSA 7-4301	66	56	56	56	56	56	56	56	56	66	61	51	46	-
DURO-TAK 87-6908	-	-	-	-	-	-	-	-	-	-	-	-	-	66
Coat weight (gsm)	~100	~250	~250	~250	~250	~250	~300	~300	~300	~350	~350	~200	~200	~50
Release liner	Fluoro-polymer coated side of Scotchpak^TM^ 1022	Uncoated side of Scotchpak^TM^ 1022												Fluoro-polymer coated side of Scotchpak^TM^ 1022
Backing membrane	Scotchpak^TM^ 9733 (polyester)	Scotchpak^TM^ 9733 (polyester)	CoTran™ 9702, 9706, 9728 (EVAC)	CoTran™ 9720 (poly-ethylene)	CoTran™ 9718 (poly-ethylene)	CoTran™ 9718 (poly-ethylene)	Silicone coated PET film (48101)	Silicone coated PET film (44916)	Silicone coated PET film (40987)	Silicone coated PET film (48101)				Scotchpak^TM^ 9733 (polyester)
Homogenization speed (rpm) and time (min) before addition of adhesive	5000; 3	30,000; 20				32,000; 30	32,000; 5							5000; 3
Homogenization speed (rpm) and time (min) after addition of adhesive	5000; 1	30,000; 1				32,000; 1	15,000; 1							5000; 1
Drying conditions	78 °C for 10 min	Air drying for 5 min. followed by drying at 78 °C for 15 min					Air drying, 15 min							78 °C for 10 min

**Table 3:** Coat weight and drug content of TAF patches.

Patch Formulation Code	COAT WEIGHT (mg/cm^2^), mean ± SD		DRUG CONTENT (mg/0.64 cm^2^), mean ± SD	
	Targeted	Experimental	Theoretical	Experimental
AP1	20	18.20 ± 1.88	0.256	0.25 ± 0.02
AP5	10	7.10 ± 0.14	0.640	0.53 ± 0.05
AP12	40	37.37 ± 6.94	0.512	0.478 ± 0.09
AP13	40	40.80 ± 1.47	0.512	0.522 ± 0.02
AP14	40	44.17 ± 4.96	0.512	0.565 ± 0.06
AP15	40	40.43 ± 2.35	0.512	0.643 ± 0.106
SP1	10	12.30 ± 3.15	0.96	1.35 ± 0.28
SP5	25	24.23 ± 2.88	2.40	2.52 ± 0.19
SP6	25	20.99 ± 5.80	2.40	2.17 ± 0.55
SP7	30	29.60 ± 1.94	2.88	2.94 ± 0.65
SP10	35	34.15 ± 3.75	1.12	1.11 ± 0.06
SP11	35	36.05 ± 1.29	2.24	2.53 ± 0.11
SP12	20	16.43 ± 0.39	2.56	2.31 ± 0.16
SP13	20	18.29 ± 1.82	3.20	3.02 ± 0.14
SP14	5	4.54 ± 0.55	0.48	0.80 ± 0.06

**Table 4:** Microscopy observations of acrylate based TAF transdermal patch formulations up to 6 months.

Patch Codes	Temperature		
	−20 °C	RT	40 °C
AP1	NO	NO	NO
AP2	NO	Crystals after three weeks	Crystals after three weeks
AP3	NO	Crystals after two weeks	Crystals after two weeks
AP4	NO	Crystals after three weeks	Crystals after three weeks
AP5	NO	Crystals after two weeks	Crystals after two weeks
AP6	Crystals after 2 months	Crystals after 9 days	Crystals after one week
AP7	Not observed	Crystals in first week	Crystals in first week
AP8	Not observed	Crystals in first week	Crystals in first week
AP9	Not observed	Smaller crystals than AP7 and AP8 after 12 days	Crystals in first week (2–3 crystals)
AP10	NO	Crystals after three weeks	Crystals after two weeks
AP11	NO	Crystals after three weeks	Crystals after two weeks
AP12	NO	NO	NO
AP13	NO	NO	NO
AP14	NO	NO	NO
AP15	NO	NO	NO

**Table 5:** Visual observations of TAF suspension patches.

Formulation Code	Properties				
	Phase Separation	Contraction/Shrinkage of Films	Residue on Release Liner after Peeling	Ease of Peeling Patches off the Skin	Residue on the Glove after Patch Removal
SP1	No	No	No	Yes	No
SP2	No	No	No	Yes	Yes
SP4	No	No	No	Yes	Yes
SP5	No	Yes (after a month)	No	Yes	Not when applied afresh, but if applied after storing for few days
SP6	No	Yes (after a month)	No	Yes	Not when applied afresh, but if applied after storing for few days
SP7	No	No	No	Yes	No
SP8	No	No	No	No	No
SP9	No	No	No	No	No
SP10	No	No	No	Yes	No
SP11	No	No	No	Yes	No
SP12	No	No	No	Yes	No
SP13	No	No	No	Yes	No
SP14	No	No	No	Yes	No

**Table 6:** Stability of TAF in different receptor solutions.

TAF Concentration (µg/mL)	% Degradation of TAF in 24 h at 37 °C			
	PBS, pH 7.4	Phosphate Buffer, pH 6.0	PEG 400: PBS, pH 7.4 (1:1)	PEG 400
0.5	22.28	3.65	41.85	Not detected due to low LOD
5	28.29	1.61	32.40	0.00
50	30.54	2.27	33.84	0.85

**Table 7:** Characterization parameters of optimized patch “SP7” up to three months at ambient and accelerated storage conditions.

Parameters	Week 1 (RT)	After 1.5 months (RT)	After 1.5 months (40 °C)	After 3 months (RT)	After 3 months (40 °C)
% TAF released after 7 days ± SE	64.27 ± 5.47	69.71 ± 5.63	66.82 ± 12.83	97.43 ± 19.03	88.86 ± 7.88
Coat weight (mg/cm2) ± SD	29.60 ± 1.94	30.38 ± 0.95	32.73 ± 3.06	29.39 ± 4.57	33.55 ± 3.14
Coat thickness (µm) ± SD	182.66 ± 1.15	186.67 ± 3.06	188.00 ± 3.46	186.00 ± 4.00	189.33 ± 3.06
Drug content (mg/cm2) ± SD	4.60 ± 1.01	4.95 ± 0.31	4.92 ± 0.39	5.25 ± 0.32	5.36 ± 0.43

## Abbreviations

ANOVA	Analysis of variance
EPI-200-SIT	EpiDerm^TM^ skin irritation test
ELISA	Enzyme linked immunosorbent assay
EVAC	Ethylene vinyl acetate copolymer
HPLC	High performance liquid chromatography
PBS	Phosphate buffered saline
PDMS	Polydimethyl-siloxane
PEG	Polyethylene glycol
PET	poly-ethylene terephthalate
PIB	Polyisobutylene
PSA	Pressure sensitive adhesives
RhE	Reconstructed human epidermis
RT	Room temperature
STRs	Single tablet regimens
TFV	Tenofovir
TAF	Tenofovir alafenamide
THF	Tetrahydrofuran
